# Management of Wilms tumor with intravenous thrombus in children: a single center experience

**DOI:** 10.1007/s12519-019-00272-0

**Published:** 2019-06-03

**Authors:** Shuai Xu, Ning Sun, Wei-Ping Zhang, Hong-Cheng Song, Cheng-Ru Huang

**Affiliations:** Department of Urology Surgery, Beijing Children’s Hospital Affiliated to Capital Medical University, National Center for Children’s Health, 56 South Lishi Road, Xicheng District, Beijing 100045, China

**Keywords:** Experience, Intravenous thrombus, Management, Wilms tumor

## Abstract

**Background:**

Wilms tumor tends to grow into vena cava, even invade atrium, which increased operating difficulty and frequency of surgical complications.

**Methods:**

Forty-two patients of Wilms tumor with intravenous thrombus were retrospective studied. The diagnosis and therapy were discussed according to the medical records and interrelated literatures.

**Results:**

Forty-two children with thrombus were diagnosed by computed tomography and 41 cases by ultrasound simultaneously. 36 children had received preoperative chemotherapy. Surgical resection was performed in all patients. Cardiopulmonary bypass was used for the removal of the intra-atrial thrombus in 5 patients. There were no surgical complications occurred. The patients received chemotherapy and radiotherapy according to clinical staging by National Wilms’ Tumor Study (NWTS)-4 or NWTS-5. 34 patients were successfully followed up, 32 patients survive at present, including one who has been followed up more than 20 years since operation.

**Conclusion:**

Standardized sequential treatment, including preoperative chemotherapy and radiotherapy, nephrectomy combining resection of thrombus, postoperative adjuvant therapy, is the mainstay of treatment of Wilms tumor with intravenous thrombus.

## Introduction

Wilms tumor is the most common urinary system malignant tumor in children. It tends to grow into vena cava, even invade into the atrium which increased operating difficulty and frequency of surgical complications [1, 2]. According to the statistics of 2731 Wilms tumor patients from 1986 to 1995 from the reports of Shamberger et al. [2], there were 165 (6%) cases with tumor thrombus, 134 (4.9%) cases with inferior vena cava tumor thrombus and 31 (1.1%) cases with tumor thrombus involving the right atrium. In literatures, there is no significant relationship between the incidence of tumor thrombus and pathological classification. The retrospective study is designed to analyze and summarize the diagnosis, treatment and prognosis of Wilms tumor children with venous tumor thrombus in our hospital.

## Methods

### General information

This is a single-center retrospective study. 42 children with unilateral Wilms tumors accompanied by intravenous tumor thrombus were enrolled: 23 males and 19 females; 16 cases in the left and 26 in the right; average age of 4.3 years. All patients were treated in urology surgery department of Beijing Children’s Hospital from January 1996 to December 2017.

### Clinical symptoms and signs

In these 42 children, there were 16 cases complained of abdominal pain, 37 cases complained of abdominal mass, and 14 cases complained of hematuria as the main symptoms for the first visiting; wherein, 2 cases had varicose veins in peripheral umbilicus during physical examination.

### Diagnostic methods

In these 42 children, the locations of tumors and tumor thrombus were clearly indicated by abdominal enhanced computed tomography (CT). Among those cases, there were 5 (12%) cases with renal vein thrombus, 27 (64%) cases with inferior vena cava thrombus (including one case whose thrombus reached the atrial entrance), and 10 (24%) cases with intra-atrial tumor thrombus. On ultrasound examination, tumor thrombus was noted in 41 children, but one case was not found. Preoperative chest X-ray and lung CT showed that 4 cases had pulmonary metastasis. One case showed adhesions of tumor to the liver on CT images, but it did not invade the liver. Only one case was confirmed by the preoperative puncture biopsy, and 41 cases were not confirmed for tumor character by biopsy, but a clinical diagnosis was made according to CT and ultrasound results. Daum et al. [3] had classified Wilms tumor thrombus into four stages: in stage I the tumor thrombus is maximal 5 cm long and reaches the inferior vena cava, ending at the lower border of the liver; in stage II the tumor mass extends under the junction of the hepatic vein; in stage III the intracaval tumor reaches the hepatic veins; and in stage IV the tumor is located in the atrium. According to Daum staging, there were 5 cases at stage I, 21 cases at stage II, 6 cases at stage III, and 10 cases at stage IV (Table 1).

**Table 1:** Effect analysis of preoperative chemotherapy in Daum stage IV tumor thrombus patients

No.	Gender	Age (y)	Tumor volumetric change	Thrombus change	CPB	Histopathology
1	Female	4.9	I	–	Yes	N
2	Male	3.5	D	–	Yes	N
3	Male	2.2	D	D	No	Mesenchymal type
4	Male	3.6	D	D	No	Germ type
5	Female	3.1	D	–	Yes	N
6	Female	2.9	D	D	No	Mixed type
7	Female	3.1	D	D	No	Mixed type
8	Female	4.8	D	–	Yes	N
9	Male	4.6	I	–	Yes	N
10	Female	3.2	D	D	No	Mesenchymal type

*CPB* cardiopulmonary bypass, *I* increase, *D* decrease, *N* necrotic tumor tissue which could not be classified after preoperative chemotherapy, – unchange

### Treatment methods

**Fig. 1:**
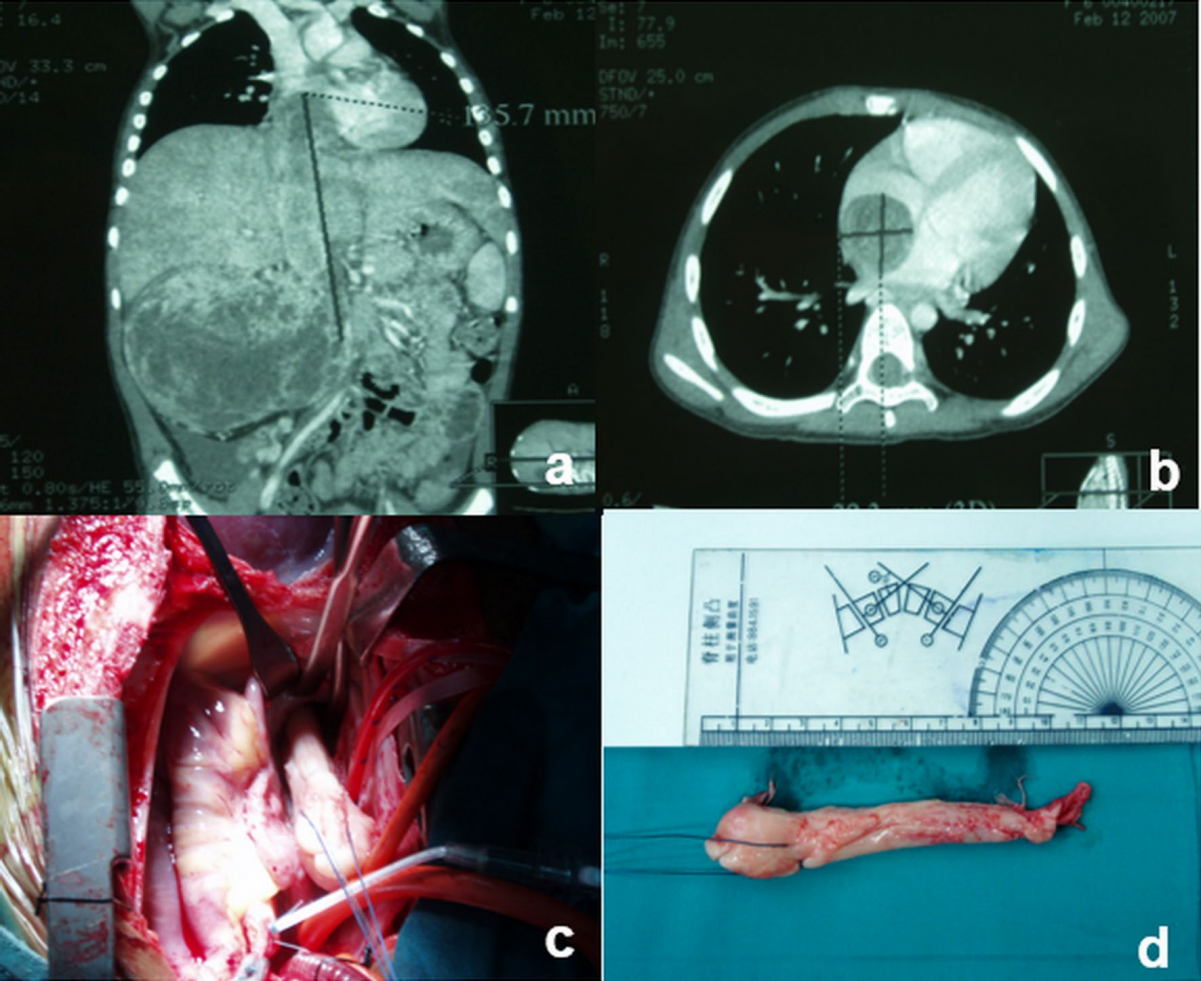
The patient (no. 8 in Table 1), female, was 4.8 years old, with right renal Wilms tumor combined with atrial tumor thrombus. She underwent deep hypothermic cardiopulmonary bypass to remove intra-cardiac tumor thrombus. **a**, **b** computed tomography showed right renal tumor and atrial tumor thrombus before preoperative chemotherapy. The tumor shrank after chemotherapy, but the intra-atrial tumor thrombus did not shrink significantly; **c**, **d** intra-atrial tumor thrombus was removed under cardiopulmonary bypass in the operation

Among these 42 Wilms tumors children with venous tumor thrombus, 36 cases underwent preoperative chemotherapy including three cases who received preoperative radiotherapy. All 42 children underwent radical nephrectomy with tumor and removal of intravenous or intra-atrial tumor thrombus. The retroperitoneal lymph nodes of 38 children resected and submitted separately for histopathologic evaluation. Because of no enlarged or hardened lymph nodes were found, the other four cases were not sent for lymph nodes pathological examination. Five cases were removed intracardiac tumor thrombus under deep hypothermic cardiopulmonary bypass (CPB) (Fig. 1). Appropriate chemotherapy and/or radiotherapy regimens were performed according to the pathological findings and National Wilms’ Tumor Study (NWTS)-4 or NWTS-5 clinical staging criteria.

## Results

**Fig. 2:**
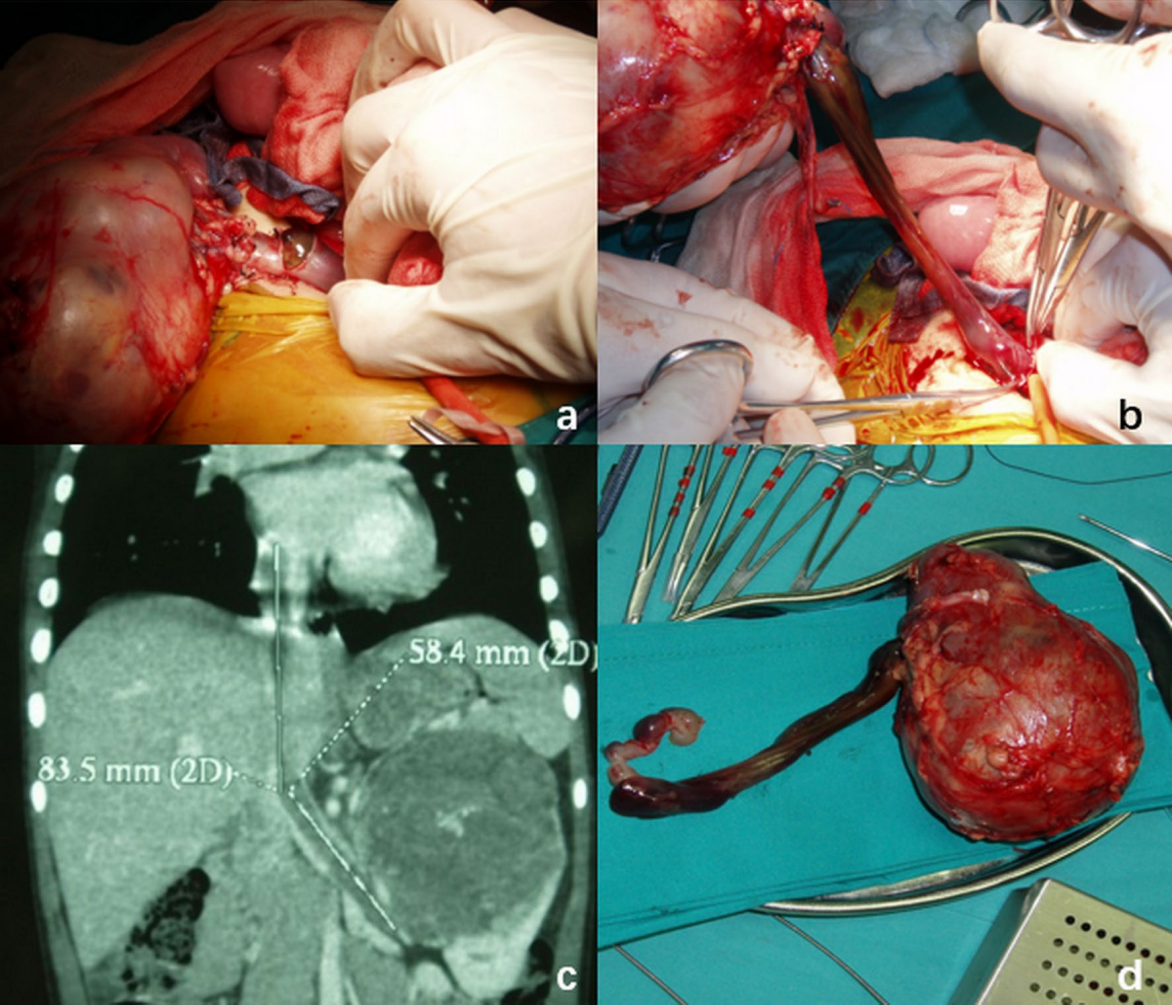
The patient (no. 4 in Table 1), male, was 3.6 years old, with left renal wilms tumor combined with atrial tumor thrombus. After preoperative chemotherapy, the tumor thrombus was significantly reduced to the atrial entrance. Preoperative ultrasound showed that there was blood flow between the thrombus and the atrial entrance or the vena cava wall. **a**, **b** during the nephrectomy, vena cava was opened carefully, tumor with the whole thrombus was completely dragged out and removed; **c** abdominal computed tomography showed left renal tumor and atrial tumor thrombus before preoperative chemotherapy; **d** specimen of the whole renal with tumor and thrombus removed by operation

Among the 42 cases, 6 cases underwent radical nephrectomy and the removal of tumor thrombus without preoperative chemotherapy. Because these tumor thrombus just reached the entrance of the inferior vena cava or within the renal vein, the nephrectomy is not difficult to perform. 36 children underwent preoperative chemotherapy with vincristine and actinomycin D for 4–8 weeks. Among the 36 cases, 3 received preoperative radiotherapy. After preoperative chemotherapy, the length of tumor thrombus shrinked in 26 cases; the size of tumor decreased in 31 cases. Especially in ten cases at Daum stage IV (Table 1), the length of tumor thrombus in five cases shrinked obviously after preoperative chemotherapy, which were out of the atrium. All children underwent radical nephrectomy and the removal of tumor thrombus, including five cases accepting CPB. 36 cases had complete removal of tumor thrombus without macroscopic residue, and 6 cases underwent segmental removal due to severe adhesions with vascular wall, with a few residues. The longest embolism was 20 cm, which entered the right atrium along the inferior vena cava; and the shortest was 1 cm, which only reached the renal vein but did not enter the inferior vena cava. One case with intra-atrial tumor thrombus, thrombus was pulled out smoothly because of no obvious adhesions between the thrombus and the vessel wall (Fig. 2). The longest operation time was 8.2 hours, and the shortest was 2.1 hours, with an average of 3.7 hours. The most intraoperative bleeding was up to 800 mL. There was no inpatient death and no postoperative complication seen.

All 42 children had the pathological type of Wilms tumor, including 18 (43%) cases of mixed type, 5 (12%) cases of mesenchymal type, 3 (7%) case of germ type, 1 (2%) case of anaplastic type, 1 (2%) case of epithelial type, and 14 (33%) cases due to necrosis after chemotherapy which could not be classified. The distribution of pathological classification and tumor thrombus stage was summarized (Table 2). Post peritoneal lymph node metastasis was confirmed in eight cases. According to NWTS-4 or NWTS-5 criteria, 20 cases were in stage II, 9 cases in stage III and 13 cases in stage IV. For stage II favorable histology (FH) Wilms tumor with a good prognosis, vincristine and actinomycin D were routinely used for 15 months (NWTS-5 used for 19 weeks). While for stage III or IV FH (or UH) Wilms tumor, adriamycin and/or cisplatin, cisplatin and/or etoposide or cyclophosphamide and/or doxorubicin were added. 22 patients received radiotherapy immediately (less than 10 days) after the operation. One patient received radiotherapy 2 years after the operation because of the recurrence of liver metastasis. 8 cases were lost to follow-up among these 42 children. Follow-up (mean follow-up time is 7.6 years) shows that 32 patients survived without tumors, out of which 20 were in clinical stage II, 8 in clinical stage III, 6 in stage IV. The longest survival time was 20 years and the shortest was 1 year. These patients included three cases with intracardiac tumor thrombus underwent excision by CPB extracorporeal circulation. two patients died of tumor recurrence. One of them had local recurrence of liver metastasis 2 years after the operation, which showed adhesions with liver during the operation and epithelial mesenchymal type was the main type, and survived with tumor until the third year after the operation. The other case with local recurrence and multiple post peritoneal lymph node metastasis died after operation 2.4 years.

**Table 2:** Distribution of pathological classification and tumor thrombus stage in the group of children (*n* = 42)

Variables	Mixed type	Epithelial type	Germ type	Mesenchymal type	Anaplastic type	*N*
Daum I	3	0	1	1	0	0
Daum II	11	1	1	2	0	6
Daum III	2	0	0	0	1	3
Daum IV	2	0	1	2	0	5

*N* necrotic tumor tissue which could not be classified after preoperative chemotherapy

Due to the large span of time, eight patients are lost to follow-up. Because of this, survival analysis was not conducted.

## Discussion

Wilms tumor with intravenous thrombus is uncommon clinically in children. Abdominal mass or abdominal circumference increase is the most common symptom of Wilms tumor. About 75–95% masses can be touched when patients first see a doctor [4]. Venous tumor thrombus is usually found concurrently when the diagnosis of Wilms tumors made. 6 of 165 children with venous tumor embolus had the corresponding manifestations such as hepatomegaly, ascites and varicose veins in the abdominal wall, accounting for only 3.6% [2]. According to the retrospective data of our hospital, only 2 patients in 42 ones was found to have varicose veins in chest and abdominal wall, accounting for 5%.

Ultrasonography has become the first choice for preliminary diagnosis of Wilms tumors and intravenous tumor thrombus for its radiationlessness, rapidity and non-invasiveness [5]. Ultrasonography has an accuracy of 60–100% in the diagnosis of tumor thrombus and can evaluate the degree of adhesions between the thrombus and vascular wall by venous blood flow. However, Khanna et al. reports that in the primary nephrectomy group, sensitivity of Doppler sonography for the detection of cavoatrial thrombus was 70.0%, lower than CT 84.6% [6]. In this study, one case with renal vein thrombus was found by enhanced CT examination, but the thrombus was found by ultrasound examination. Based on our experience, if vena cava is widened and enlarged or a low-density filling-defect is shown by CT, tumor thrombus may exist. Enhanced CT and magnetic resonance imaging are more sensitive, which can accurately diagnose the intravenous and intra-atrial tumor thrombus and can show the location, size of the thrombus and the relationship with the vascular wall. They are helpful to judging the timing of operation, the choice of the operation methods and the judgement whether the tumor thrombus can be pulled out completely. Although inferior vena cava angiography has a certain diagnostic value, there are risks of the tumor thrombus falling off and embolism when using this examination. Preoperative chest X-ray should be a routine examination and lung CT may be used to determine the presence or absence of metastasis if necessary.

Intravenous thrombus caused by Wilms tumor, which exceed the level of hepatic vein, especially when the thrombus reaches the atrium or the primary tumors are too large to be completely excised, preoperative chemotherapy is recommended. It can reduce the size of tumor and thrombus, probability of tumor rupture during the operation and the usage rate of CPB, death rate and surgical complications are also reduced [7]. SIOP-9 (The International Society of Pediatric Oncology in Europe) study found that preoperative chemotherapy for 4 weeks and 8 weeks was equally effective in improving the prognosis of children [8]. In our hospital, we recommend preoperative chemotherapy with vincristine and actinomycin D for 4–8 weeks. The study of Shamberger et al. showed that during the operation, 61% of the patients who did not receive preoperative chemotherapy were found to have adhesions between the thrombus and the vessel wall, and the children receiving preoperative chemotherapy accounted for 44% [2]. In our study, 86% patients underwent preoperative chemotherapy. Most cases were sensitive to chemotherapy: 72% thrombus shrinked and 85% tumor decreased in preoperative chemotherapy cases. Especially in 10 cases with the atrium thrombus (Daum stage IV), thrombus in half of these cases shrinked out of the atrium (Table 1). Benefiting from preoperative chemotherapy, the using of CPB was reduced. Only five cases need CPB to remove atrium thrombus. Preoperative chemotherapy significantly reduced complications associated with CPB including infection, thrombus rupture, and bleeding. Interestingly, it is not both tumor and tumor thrombus being sensitive for chemotherapy in Daum stage IV patients. Only five cases of tumor and tumor thrombus decreased simultaneously. There are no relevant reports. We think that the reason may be the obvious adhesion between the atrial tumor thrombus and the blood vessel wall. Even if the tumor thrombus is sensitive to chemotherapy, its shrinkage may be limited, which needs to be further studied.

In this study, tumor thrombus was not sensitive to preoperative chemotherapy in ten patients, including five patients with Daum IV. The pathological type was: mesenchymal type (*n* = 3), epithelial type (*n* = 1), mixed type (*n* = 1) and necrotic tumor tissue (*n* = 5). Pathologically, solid mature type tumor tissues, such as mesenchymal type, epithelial type or fetal rhabdomyoma type Wilms tumors, were not sensitive to preoperative chemotherapy, compared with germinal type tumors. However, children with these pathological types usually have a good prognosis after surgery and postoperative chemotherapy and/or radiotherapy.

However, NWTS researchers believed that preoperative chemotherapy for Wilms tumor may interfere with the histopathologic type, affect the detection rate of anaplasia, reduce the clinical stage, and may miss the diagnosis of bilateral Wilms tumor, and will thus affect the prognosis. It was found that for some children with Wilms tumor without lymph node metastasis, which were evaluated as stage II after the preoperative chemotherapy, the recurrence rate of tumor was higher than that those without preoperative chemotherapy [9].

Tumor and thrombus removal are the basis of surgical treatment in Wilms tumor with intravenous thrombus in children. For children with no shrinkage or progression of tumors after preoperative chemotherapy, surgery should be performed as soon as possible [10, 11]. The surgical strategy we recommended as follows: for Daum stage I–II tumor thrombus, the renal artery was ligated after exposure of the renal pedicle, and the vena cava was blocked by the vascular clamps at both ends of the tumor thrombus when exposed along the renal vein. The renal vein was cut off, and the tumor thrombus was pulled first. If the thrombus could have been removed completely, then the renal vein was ligated. For the tumor thrombus infiltrating and growing into the blood vessel wall, a T-shaped incision of the vena cava can be made to separate the tumor thrombus with a brain dissector to avoid injury to the vena cava as far as possible, and the common incision of the vena cava can be sutured in situ. For the thrombus at stage III–IV, especially stage IV, CPB should be used by cooperating with the cardiothoracic surgeon. The thrombus should be removed gently to avoid pulmonary embolism due to the breakup and shedding of the tumor [12]. In our study group, five children with intracardiac tumor thrombus were operated on under CPB with hypothermia. There was no hospital mortality and postoperative complication in the five patients. Follow-up showed that one child died as a result of liver metastases after operation 3.2 years. Chiappini et al. [13] reported that 13 patients who underwent deep hypothermic CPB for resection of intracardiac tumor thrombus had a hospital mortality rate of 0, with postoperative complications mainly including respiratory failure and hemorrhage. After a follow-up for 33.9 months, 8 (62%) patients survived. There is no evidence that the recurrence rate increases in patients with intra-operative remnant tumors in vessel wall. In our study group, six cases underwent segmental removal with a few residues due to severe adhesions with vascular wall. Among these six cases, tumor thrombus in five cases was not sensitive to preoperative chemotherapy. Postoperative chemotherapy was continued, and no recurrence was observed during chemotherapy. However, follow-up show that 1 case died due to the recurrence of the tumor in situ and multiple post peritoneal lymph node metastasis after operation 2.4 years; four of the other five cases survived without tumor till now, and one case lost follow-up due to long time term.

Pathological type of the group was shown as follows: 18 cases of mixed type, 5 cases of mesenchymal type, 3 case of germ type, 1 case of anaplastic type, 1 case of epithelial type, and 14 cases due to necrosis after chemotherapy which could not be classified. The largest number of pathological types was mixed types, and most were distributed in Daum stage II. One anaplastic type case belongs to Daum stage III, which died as a result of multiple post peritoneal lymph node metastasis as the article had mentioned. Because most of patients underwent preoperative chemotherapy, the pathology of 14 cases showed necrosis could not be classified. Three germ type cases were distributed in Daum stage I, II and IV, in which stage I and II were survived free of tumor till now, and the follow-up times are 6.2 and 3 years after operation, respectively. However, the stage IV patient was lost to follow-up.

Appropriate radiotherapy and chemotherapy were delivered according to pathological classification and clinical stage after the operation. According to the latest NWTS staging, the patients who receive any biopsy before resection of the tumor, those undergoing debris resection including separate removal of the thrombus in the vein, resection margins microscopically or macroscopic incomplete excision as well as tumor rupture before or intra-operatively including spillage confined to the flank or diffuse peritoneal contamination by the tumor or where peritoneal implants are present, are classified as those at stage III and require postoperative radiotherapy [14]. NWTS-5 pointed out that patients in stage III with non-anaplastic histopathology, vincristine, actinomycin D and adriamycin supplemented by abdominal radiotherapy are the best postoperative treatment options. Even if the cumulative dosage of adriamycin reaches 150 mg per m^2^, the incidence of long-term cardiotoxicity is still low. Although the long-term complications of radiotherapy may seriously affect the quality of life of the children and even cause death, postoperative radiotherapy still plays an important role in improving the prognosis of children with a poor prognosis by pathological classification and who are in stage III–V [15]. A retrospective study of NWTS showed that radiotherapy within 10 days after operation was important for improving the prognosis. How to control the dosage of radiotherapy and reduce the radiotherapy-related complications and mortality while improving the survival rate still requires constant experience summary.

NWTS-4 reported that the 2-year survival rate of children with Wilms tumor was 91%. There was no significant difference in 3-year survival rate between the patients with venous or intra-atrial tumor thrombus and those without tumor thrombus in the same pathological type and stage. The percent of patients with a good and poor prognosis were 90% and 41.7%, respectively, while the values of patients at stage II, III and IV were 100, 94.7 and 75.3%, respectively [2]. Because there is basically no difference in prognosis, it is more important to minimize the surgical complications and the long-term harm caused by chemotherapy and radiotherapy.

In conclusion, comprehensive sequential treatment should be adopted for children with Wilms tumor accompanied by intravenous tumor thrombus. Preoperative chemotherapy should be performed for those with a higher surgical risk from larger tumors and thrombus to reduce the difficulty and complications of operation. Surgical removal of the tumors and tumor thrombus is the key to a good prognosis. Postoperative radiotherapy plays an important role in improving the prognosis of children who are above clinical stage II (stage III–IV), and it should be added within 10 days after operation. Standardized comprehensive treatment may enable most portions of children to survive for a long time.

## References

[1] Luck SR, DeLeon S, Shkolnik A, Morgan E, Labotka R (1982). Intracardiac Wilms’ tumor: diagnosis andmanagement. J Pediatr Surg.

[2] Shamberger RC, Ritchey ML, Haase GM, Bergemann TL, Loechelt-Yoshioka T, Breslow NE (2001). Intravascular extension of Wilms tumor. Ann Surg.

[3] Daum R, Roth H, Zachariou Z (1994). Tumor infiltration of the vena cava in nephroblastoma. Eur J Pediatr Surg..

[4] Exelby PR (1981). Retroperitoneal malignant tumors: wilms’ tumor and neuroblastoma. Surg Clin N Am.

[5] Horan JJ, Robertson CN, Choyke PL, Frank JA, Miller DL, Pass HI (1989). The detection of renal cell carcinoma into the renal vein and inferior vena cava: a prospective comparison of venography and magnetic resonance imaging. J Urol..

[6] Khanna G, Rosen N, Anderson JR, Ehrlich PF, Dome JS, Gow KW (2012). Evaluation of diagnostic performance of CT for detection of tumor thrombus in children with Wilms tumor: a report from the Children’s Oncology Group. Pediatr Blood Cancer.

[7] Cristofani LM, Duarte RJ, Almeida MT, Odone Filho V, Maksoud JG, Srougi M (2007). Intracaval and intracardiac extension of Wilms’ tumor. The influence of preoperative chemotherapy on surgical morbidity. Int Braz J Urol..

[8] Lall A, Pritchard-Jones K, Walker J, Hutton C, Stevens S, Azmy A (2006). Wilms’ tumor with intracaval thrombus in the UK Children’s Cancer Study Group UKW3 trial. J Pediatr Surg.

[9] Tournade MF, Com-Nougué C, Voûte PA, Lemerle J, de Kraker J, Delemarre JF (1993). Results of the Sixth International Society of Pediatric Oncology Wilms’ Tumor Trial and Study: a risk-adapted therapeutic approach in Wilms’ tumor. J Clin Oncol.

[10] Berberoğlu S, Akyüz C, Büyükpamukçu M (1996). Successful treatment of intracaval and atrial extension of Wilms’ tumour by chemotherapy. Postgrad Med J.

[11] Cox SG, Davidson A, Thomas J, Brooks A, Hewitson J, Numanoglu A (2018). Surgical management and outcomes of 12 cases of Wilms tumor with intracardiac extension from a single centre. Pediatr Surg Int.

[12] Murthi GV, Kocyildirim E, Sellathury S, Cuckow PM, Wilcox DT, Michalski A (2006). Wilms’ tumour with persistent intravascular extension: a review of the surgical aspects of management. J Pediatr Urol..

[13] Chiappini B, Savini C, Marinelli G, Suarez SM, Di Eusanio M, Fiorani V (2002). Cavoatrial tumor thrombus: single-stage surgical approach with profound hypothermia and circulatory arrest, including a review of the literature. J Thorac Cardiovasc Surg.

[14] Bartlett DL, Thirunavukarasu P, Neal MD, Bao P, Pragatheeshwar KD (2010). Surgical oncology: fundamentals, evidence-based approaches and new technology.

[15] She JB, Liu TB, Xu Z, Li SS (2004). Study on long-term complications of nephroblastoma after radiotherapy. J SUN Yat-sen Univ (Med Sci)..

